# High prevalence and pathogenic potential of Shiga toxin-producing *Escherichia coli* strains in raw mutton and beef in Shandong, China

**DOI:** 10.1016/j.crfs.2022.08.021

**Published:** 2022-09-15

**Authors:** Bin Hu, Xi Yang, Qian Liu, Yuanqing Zhang, Deshui Jiang, Hongbo Jiao, Ying Yang, Yanwen Xiong, Xiangning Bai, Peibin Hou

**Affiliations:** a Shandong Center for Disease Control and Prevention, Jinan, 250014, Shandong, China; b State Key Laboratory of Infectious Disease Prevention and Control, National Institute for Communicable Disease Control and Prevention, Chinese Center for Disease Control and Prevention, Beijing, 102206, China; c Lanling Center for Disease Control and Prevention, Lanling, 277700, Shandong, China; d Division of Clinical Microbiology, Department of Laboratory Medicine, Karolinska Institute, 141 52, Stockholm, Sweden

**Keywords:** Shiga toxin-producing *Escherichia coli*, Raw meats, Beef, Mutton, Contamination, Whole genome sequencing

## Abstract

Shiga toxin-producing *Escherichia coli* (STEC) is a foodborne pathogen that can cause severe human diseases such as hemolytic uremic syndrome (HUS). Human STEC infections are frequently caused through consumption of contaminated foods, especially raw meats. This study aimed to investigate the prevalence of STEC in raw meats and to characterize the meat-derived STEC strains using whole genome sequencing. Our study showed that 26.6% of raw mutton, and 7.5% of raw beef samples were culture-positive for STEC. Thirteen serotypes were identified in 22 meat-derived isolates in this study, including the virulent serotypes O157:H7 and O26:H11. Seven Shiga toxin (Stx) subtypes were found in 22 isolates, of these, *stx1c* and *stx1c* + *stx2b* were predominant. The recently-reported *stx2k* subtype was found in three mutton-sourced isolates. A number of other virulence genes such as genes encoding intimin (*eae*), enterohemorrhagic *E. coli* (EHEC) hemolysin (*ehxA*), EHEC factor for adherence (*efa1*), heat-stable enterotoxin 1 (*astA*), type III secretion system effectors, were detected in meat-derived STEC strains. One mutton-sourced isolate was resistant to three antibiotics, i.e., tetracycline, chloramphenicol, and trimethoprim-sulfamethoxazole. Whole-genome phylogeny indicated the genomic diversity of meat-derived strains in this study. O157:H7 and O26:H11 isolates in this study were phylogenetically grouped together with strains from HUS patients, suggesting their pathogenic potential. To conclude, our study reported high STEC contaminations in retail raw meats, particularly raw mutton, genomic characterization indicated pathogenic potential of meat-derived STEC strains. These findings highlight the critical need for increased monitoring of STEC in retail raw meats in China.

## Highlights

• High prevalence of Shiga toxin-producing *E. coli* (STEC) was detected in raw mutton, compared to beef.
• Virulent serotypes O157:H7 and O26:H11 were found in meat-sourced STEC isolates.
• Meat-sourced STEC isolates in the same region exhibited genetic diversity.

## Graphical abstract

**Figure:**
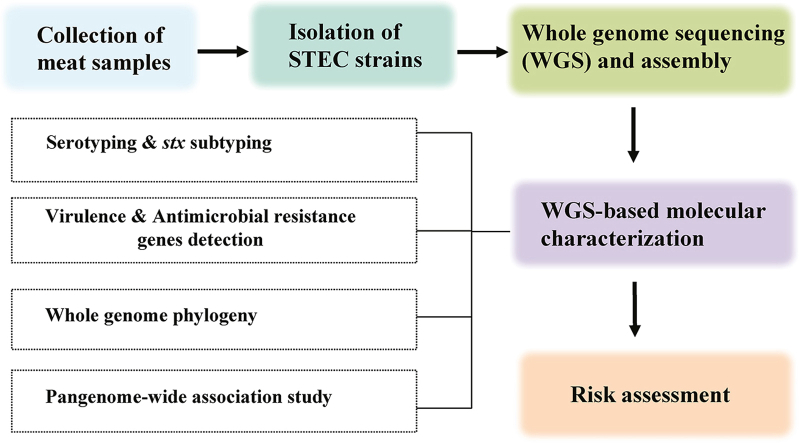

## 1 Introduction

Shiga toxin–producing *Escherichia coli* (STEC) is an important foodborne pathogen, which can cause human diseases ranging in severity from asymptomatic carriage to non-bloody/bloody diarrhea (BD) and even fatal hemolytic uremic syndrome (HUS) (Bryan et al., 2015). O157:H7 has been the predominant serotype associated with severe clinical outcome such as HUS (Fatima and Aziz, 2022). In recent years, non-O157 STEC serogroups have grown in importance due to their increasing incidence and ability to cause mild to severe diseases, in particular serogroups O26, O45, O103, O111, O121, and O145, referred to as the top six non-O157 STEC (Smith et al., 2014; Valilis et al., 2018).

STEC features a broad spectrum of virulence determinants, with the primary disease-causing factor being the Shiga toxin (Stx). Stx was classified into two immunologically distinct types, Stx1 and Stx2, which can be further divided into several Stx1/Stx2 subtypes (Scheutz et al., 2012). Subtypes Stx2a, Stx2c, and Stx2d are associated with severe human illnesses such as HUS, and Stx1 is frequently implicated in mild illness such as non-bloody diarrhea (Bai et al., 2021; Koutsoumanis et al., 2020). The emergence of new subtypes and their clinical relevance, e.g., Stx2k (Yang et al., 2020), Stx2m (Bai et al., 2021), highlighted the pathogenic potential of STEC strains producing new Stx subtypes. STEC encodes other virulence factors involved in the pathogenic process, including adhesins, toxins, secretion system, and others (Bryan et al., 2015). The principal adherence factor in STEC is the intimin encoded by the *eae* gene on the locus of enterocyte effacement (LEE) pathogenicity island. Intimin contributes to the intimate adherence to enterocytes and formation of the attaching and effacing intestinal lesions (Kaper and O'Brien, 2014). STEC strains carrying both the *eae* and *stx2* genes are more strongly correlated with severe clinical symptoms such as HUS (Hua et al., 2020). LEE-negative STEC strains can also cause disease through other mechanisms of intestinal attachment (Montero et al., 2019). Various fimbrial and nonfimbrial adhesin-encoding genes have been reported in LEE-negative STEC strains, e.g., *efa1* (enterohemorrhagic *E. coli* factor for adherence), *paa* (porcine attaching and effacing associated), *iha* (*Vibrio cholerae* IrgA homolog), *ompA* (outer membrane protein A), *lpfA* (long polar fimbriae), *fimA* (type 1 fimbriae), and a recently-described gene *hes* (hemagglutinin from Shiga toxin-producing *E. coli*) located on the locus of adhesion and autoagregation (LAA) (McWilliams and Torres, 2014; Montero et al., 2019; Toma et al., 2004; Velez et al., 2020). Other virulence factors that may play a role in STEC pathogenesis included the enterohemorrhagic *E. coli* (EHEC) hemolysin encoded by EHEC-*hlyA* (*ehxA*) gene (Bielaszewska et al., 2014), which may contribute to the hemolytic activities of STEC strains. In addition, some STEC strains possess the gene *astA* encoding enteroaggregative *E. coli* (EAEC) heat-stable enterotoxin, which has been shown to be associated with severe clinical outcome (Bai et al., 2021).

Ruminants, such as cattle and sheep, are the most important reservoirs of STEC (Gyles, 2007; McCarthy et al., 2021). Previous source attribution studies have indicated that domestic ruminants account for approximately three-quarters of reported human STEC infections, and that consumption of beef and beef product is a significant risk factor for human infection with STEC attributed to cattle (Kosmider et al., 2010; Mughini-Gras et al., 2018). Although human STEC cases attributed to sheep are not as frequently reported as those attributed to cattle, accumulating data show high prevalence of STEC in sheep and mutton (Bai et al., 2015; Kumar et al., 2014; McCarthy et al., 2021). An outbreak of HUS caused by STEC in Norway has been traced to contaminated mutton (Schimmer et al., 2008), confirming that consumption of raw/undercooked meat of any origin can be an important source of human STEC infection. In China, various studies have shown STEC contaminations in raw meats (Bai et al., 2015; Chao et al., 2007; Dong et al., 2020; Li et al., 2011; Zhou et al., 2002). Of note, a recent study reported multidrug resistant STEC strains with high pathogenic potential from retail beef in China (Hu et al., 2021), this knowledge is limited for mutton-derived strains. The objective of this study was to depict the prevalence, genomic and antimicrobial characteristics of STEC strains in retail raw meats with a particular interest on mutton in Jinan, Shandong, China, and to assess their pathogenic potential.

## 2 Materials and methods

### 2.1 Sample collection and strain isolation

A total of 131 samples of raw meat, including 64 raw mutton and 67 raw beef were purchased in Jinan city, Shandong, China, between 2018 and 2019. Only one sample per retail meat market stall was collected. STEC strains were isolated using the methods described previously with minor modification (Bai et al., 2015). Briefly, meat samples were enriched in EC broth (Beijing Landbridge Technology Co., Ltd., China), and incubated overnight at 37 °C. Given the small sample size, all enriched samples were inoculated into two selective media CHROMagar™ ECC agar and CHROMagar™ STEC agar (CHROMagar, France) for isolation of STEC strains as described previously (Bai et al., 2015). After overnight incubation at 37 °C, presumptive colonies were picked and tested for *stx* genes by single colony duplex PCR assay. API 20E biochemical test strips (bioMérieux, France) were used for confirmatory test. To capture O157 STEC, immunomagnetic separation (IMS) with magnetic beads coated with antibody to O157 (Tianjin Biochip Co., Ltd., China) was performed with the enrichment of *stx*-positive samples according to the manufacturer's protocol, the concentrated samples were inoculated onto the two selective media and following steps were repeated as described above. Only one isolate per sample was kept for further analysis.

### 2.2 Genome sequencing and assembly

Genomic DNA of each STEC isolate was extracted from an overnight culture using the Wizard Genomic DNA purification kit (Promega, USA) according to the manufacturer's instructions. Sequencing library was constructed using NEBNext® Ultra™ DNA Library Prep Kit for Illumina (NEB, USA) following manufacturer's recommendations, and index codes were added to attribute sequences to each sample. Whole genome sequencing was performed using Illumina NovaSeq 6000 platform at the Beijing Novogene Bioinformatics Technology Co., Ltd., China. Illumina PCR adapter and low-quality reads (quality scores ≤20) were filtered using readfq (version 10). The filtered-reads were assembled into scaffolds using the SOAPdenovo (Li et al., 2008; Li et al., 2010).

### 2.3 Determination of serotype, *stx* subtype, virulence factor genes, antimicrobial resistance genes, and sequence type

The genome assemblies of all STEC isolates were subjected to our whole genome analysis pipelines to characterize the genomic features including serotypes, *stx* subtypes, virulence genes and antimicrobial resistance genes as recently described (Bai et al., 2021; Yang et al., 2022; Yang et al., 2021). Briefly, the EcOH database (https://github.com/katholt/srst2/blob/master/data/EcOH.fasta), VFDB database (http://www.mgc.ac.cn/VFs/main.htm), and Comprehensive Antibiotic Resistance Database (CARD) (https://card.mcmaster.ca), were used to determine the serotypes, virulence genes and antimicrobial resistance genes, respectively, using ABRicate version 1.0.1 (https://github.com/tseemann/abricate) with default parameters. For *stx* subtyping, an *in-house stx* subtyping database was created with ABRicate by integrating representative nucleotide sequences of all identified *stx1* and *stx2* subtypes, consisting of *stx1*/*stx2* subtypes previously reported by Scheutz et al. (2012), and several recently-reported Stx2 subtypes, Stx2h-Stx2m and Stx2o (Bai et al., 2021; Gill et al., 2022). The assemblies were then compared against the *in-house stx* subtyping database using ABRicate version 1.0.1. Multi-locus sequence typing (MLST) was conducted *in silico* using the on-line tool provided by the Warwick *E. coli* MLST scheme website (https://enterobase.warwick.ac.uk/species/ecoli/allele_st_search).

### 2.4 Antimicrobial susceptibility testing

The minimal inhibitory concentrations of all STEC isolates were performed using broth microdilution method as previously described (Pan et al., 2021). The qualitative interpretations of susceptible (S), intermediate (I), resistant (S) strains were determined according to the standard of the Clinical Laboratory Standards Institute guidelines (CLSI 2020). Nineteen antimicrobial agents were tested in this study. These included ampicillin (2–32 g/mL), amikacin (4–64 g/mL), ampicillin-sulbactam (1–32 g/mL), azithromycin (2–64 g/mL), aztreonam (0.25–16 g/mL), cefoxitin (2–64 g/mL), ciprofloxacin (0.015–2 g/mL), ceftazidime-avibactam (0.25/4–8/4 g/mL), cefotaxime (0.25–16 g/mL), ceftazidime (0.25–16 g/mL), colistin (0.25–8 g/mL), chloramphenicol (4–32 g/mL), ertapenem (0.25–8 g/mL), imipenem (0.25–8 g/mL), meropenem (0.125–8 g/mL), nalidixic acid (4–32 g/mL), nitrofurantoin (32–256 g/mL), tetracycline (1–16 g/mL), and trimethoprim-sulfamethoxazole (0.5–8 g/mL).

### 2.5 Phylogenetic analysis

Whole-genome multilocus typing (wgMLST) and whole-genome phylogeny analysis were performed to assess phylogenic relationships of STEC isolates. Given the particular focus on the mutton-derived STEC strains in this study, strains from mutton previously collected in China, together with four clinical STEC strains from HUS patients (two O157:H7 and two O26:H11 genomes) were included in the analyses. The complete whole-genome sequence of O157:H7 strain Sakai (NC_002695.2) was used as a reference genome. An *ad hoc* fast-GeP analysis (https://github.com/jizhang-nz/fast-GeP) (Zhang et al., 2018) was used to define wgMLST allelic profiles. Whole-genome phylogeny was inferred from concatenated sequences of all shared loci using Gubbins (version 2.3.4) with default settings (Croucher et al., 2015). Single Nucleotide Polymorphism (SNP)-based phylogeny and the SNP distance were obtained by using snippy-multi in Snippy version 4.3.6 (https://github.com/tseemann/snippy) and snp-dists v0.7.0 (https://github.com/tseemann/snp-dists) with the default parameters.

### 2.6 Pangenome-wide association study

The pangenomes of STEC isolates in this study and reference mutton-derived STEC isolates reported previously were calculated from the harmonized genome annotations produced by Prokka using Roary (https://github.com/sanger-pathogens/Roary) (Page et al., 2015). The accessory genes were associated to the source of isolates using Scoary v1.6.16 (run with 1000 permutation replicates) (Brynildsrud et al., 2016). Accessory genes were reported as statistically significantly associated to a variable if they attained a Benjamini-Hochberg corrected *p*-value below 0.05. Multiple correspondence analysis (MCA) of pangenomes was performed using the gene presence/absence table generated from Roary as previously described (Bai et al., 2021). The R function MCA from R package FactoMineR was used for the analysis (Lê et al., 2008).

### 2.7 Data availability

The draft genomes of 22 STEC isolates in this study were deposited in GenBank under the accession numbers shown in Fig. 1.

**Fig. 1:**
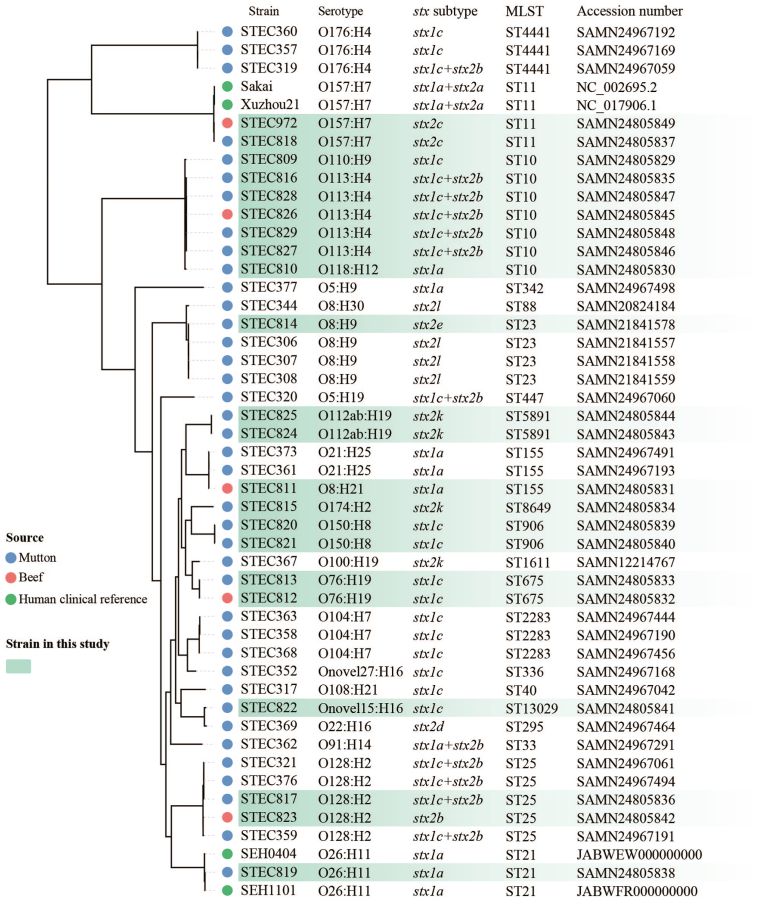
**Whole-genome phylogeny of Shiga toxin-producing *Escherichia coli* (STEC) isolates.** Strain, serotype, *stx* subtype, MLST, and accession number of all STEC isolates are shown. The source of isolates, i.e., mutton, beef, and human clinical reference O157:H7 and O26:H11 isolates, are marked as indicated. Isolates from raw meats in this study are highlighted in green shadow. (For interpretation of the references to colour in this figure legend, the reader is referred to the Web version of this article.)

## 3 Results

### 3.1 Occurrence of STEC in raw mutton and beef

Out of 131 samples of raw meats collected between 2018 and 2019 from retail market in Jinan city, 22 samples (16.8%) were culture-positive for STEC strains. The culture-positive rate of STEC in raw mutton was ∼3 times higher than that of beef, in particular, 36% of mutton samples collected in 2019 were culture-positive for STEC (Table 1).

**Table 1:** Prevalence of STEC strains in raw mutton and beef.

Year	Raw mutton			Raw beef		
	No. of samples	No. of STEC isolates	Culture positive (%)	No. of samples	No. of STEC isolates	Culture positive (%)
2018	39	8	20.5	25	2	8
2019	25	9	36	42	3	7.1
Total	64	17	26.6	67	5	7.5

### 3.2 Molecular characteristics of STEC isolates from raw meats

Thirteen serotypes were identified among 22 STEC isolates (Table 2). The most predominant serotype was O113:H4, comprising of four mutton-sourced isolates and one beef-sourced isolate. O157:H7 was identified in one mutton-sourced isolate and one beef-sourced isolate, O26:H11 was identified in one mutton-sourced isolate. Seven *stx* subtypes/combinations were found in all isolates, of these, *stx1c* and *stx1c* + *stx2b* were most predominant comprising of 6 isolates each. The two O157:H7 isolates carried *stx2c*, and the O26:H11 isolate carried *stx1a*. Of note, the recently-identified *stx2k* subtype (Yang et al., 2020) was found in three mutton-sourced isolates, two *stx2k*-carrying isolates were assigned to O112ab:H19 serotype, the remaining one was O174:H2, and the three sequences of *stx2k* were identical to *stx2k* carried by the reference patient-derived Stx2k-STEC (strain ID STEC309). One isolate from mutton carried *stx2e* subtype.

**Table 2:** Characteristics of 22 STEC isolates from raw meats in this study.

Strain ID	Source	Serotype	*stx* subtype	MLST	Main virulence genes^a^
STEC809	mutton	O110:H9	*stx1c*	10	*astA*, *ompA*, *fimA*
STEC810	mutton	O118:H12	*stx1a*	10	*astA*, *ompA*
STEC811	beef	O8:H21	*stx1a*	155	*ompA*, *fimA*
STEC812	beef	O76:H19	*stx1c*	675	*ehxA*, *ompA*, *fimA*
STEC813	mutton	O76:H19	*stx1c*	675	*ehxA*, *ompA*, *fimA*
STEC814	mutton	O8:H9	*stx2e*	23	*ompA*, *fimA*
STEC815	mutton	O174:H2	*stx2k*	13029	*ompA*, *fimA*
STEC816	mutton	O113:H4	*stx1c* + *stx2b*	10	*ehxA*, *astA*, *ompA*, *fimA*
STEC817	mutton	O128:H2	*stx1c* + *stx2b*	25	*ehxA, ompA*, *fimA*
STEC818	mutton	O157:H7	*stx2c*	11	*eae*, *ehxA*, *astA*, *paa*, *ompA*, *fimA*
STEC819	mutton	O26:H11	*stx1a*	21	*eae*, *ehxA*, *efa1*, *ompA*, *fimA*
STEC820	mutton	O150:H8	*stx1c*	906	*ehxA*, *ompA*, *fimA*
STEC821	mutton	O150:H8	*stx1c*	906	*ehxA*, *ompA*, *fimA*
STEC822	mutton	Onovel15:H16	*stx1c*	8649	*ompA*, *fimA*
STEC823	beef	O128:H2	*stx2b*	25	*ehxA*, *ompA*, *fimA*
STEC824	mutton	O112ab:H19	*stx2k*	5891	*ompA*, *fimA*
STEC825	mutton	O112ab:H19	*stx2k*	5891	*ompA*, *fimA*
STEC826	beef	O113:H4	*stx1c* + *stx2b*	10	*ehxA*, *astA*, *ompA*, *fimA*
STEC827	mutton	O113:H4	*stx1c* + *stx2b*	10	*ehxA*, *astA*, *ompA*, *fimA*
STEC828	mutton	O113:H4	*stx1c* + *stx2b*	10	*ehxA*, *astA*, *ompA*, *fimA*
STEC829	mutton	O113:H4	*stx1c* + *stx2b*	10	*ehxA*, *astA*, *ompA*, *fimA*
STEC972	beef	O157:H7	*stx2c*	11	*eae*, *ehxA*, *astA*, *paa*, *ompA*, *fimA*

a The presence of virulence genes *eae*, *ehxA, efa1*, *paa*, *astA*, *ompA*, and *fimA* is shown in this table, the presence of other virulence genes is shown in Supplementary Table S1.

Besides *stx1*/*stx2*, a number of virulence genes were detected in 22 meat-derived STEC isolates (Table 2 and Supplementary Table S1). The intimin encoding gene *eae* was present in the two O157:H7 isolates and the mutton-sourced O26:H11 isolate. The two O157:H7 isolates also harbored other adherence genes *paa*, *ompA*, and *fimA*. O26:H11 isolate possessed *efa1*, *ompA*, and *fimA*. EHEC hemolysin gene *ehxA* was present in 14 STEC isolates, including two O157:H7 isolates, one O26:H11, and 11 other non-O157 STEC isolates. The heat-stable enterotoxin 1 encoding gene *astA* was found in two O157:H7 isolates and other seven non-O157 STEC isolates. Other virulence factors identified in STEC isolates mainly included type III secretion system effectors, fimbrial proteins, etc. (Supplementary Table S1). No statistical difference in virulence genes was found between mutton- and beef-sourced STEC isolates.

### 3.3 Antimicrobial resistance of meat-derived STEC isolates

Among the 19 antibiotics tested in this study, all isolates were susceptible to 15 antibiotics including amikacin, ampicillin, ampicillin-sulbactam, azithromycin, aztreonam, cefotaxime, cefoxitin, ceftazidime, ciprofloxacin, ertapenem, imipenem, meropenem, nalidixic acid, nitrofurantoin, and ceftazidime-avibactam. One mutton-sourced isolate (strain ID STEC809) was resistant to three antibiotics, i.e., tetracycline, chloramphenicol, and trimethoprim-sulfamethoxazole. One mutton-sourced isolate (strain ID STEC810) was resistant to tetracycline. A number of antimicrobial resistance genes were detected in 22 STEC isolates (Supplementary Table S2). Isolates that were resistant to certain antibiotics carried corresponding resistant genes. For instance, strain STEC809 carried genes involved in resistance to chloramphenicol (*floR*), tetracycline (*tet(A)*, *emrK*, *emrY*), and trimethoprim-sulfamethoxazole (*dfrA17*, *sul2*). Strain STEC810 resistant to tetracycline carried corresponding resistant gene *tet(A)*.

### 3.4 Whole genome phylogeny and pangenome-wide association study

A whole-genome phylogenetic tree was constructed from alignment of concatenated sequences of the 3209 shared-loci found in 48 STEC genomes, including 22 isolates in this study, 22 mutton-derived isolates previously collected from China (Bai et al., 2015), two O157:H7 and two O26:H11 genomes of strains from HUS patients downloaded from GenBank (Fig. 1). The two O157:H7 isolates (one from beef and one from mutton) in this study were phylogenetically grouped together with two outbreak O157:H7 strains, and the mutton-sourced O26:H11 isolate was grouped together with two O26:H11 strains from HUS patients in Sweden, indicating the pathogenic potential of these meat-derived strains. We observed that isolates with same serotype or *stx* subtype were more likely to cluster together. Two mutton-derived STEC isolates in this study (strain ID STEC817 and STEC814) shared the same serotype, *stx* subtypes, and phylogenetically clustered with mutton-sourced STEC isolates previously collected China. Interestingly, a few isolates with dissimilar serotypes were grouped closely. For instance, one beef-sourced isolate (strain ID STEC811, serotype O8:H21) and two previously reported mutton-sourced isolates (strain ID STEC361 and STEC373, serotype O21:H25) were grouped together (Fig. 1). To confirm their genetic relatedness, we performed SNP analysis on the three isolates together with two O112ab:H19 isolates (strain ID STEC824 and STEC825) that were grouped closely on the phylogenetic tree, the SNP distances among the three strains were ≤119 (Supplementary Fig. S1).

Pangenome-wide study was further performed with attempt to identify any association between accessory genes and strain classification. A total of 13,843 genes were found in pangenomes of 44 meat-derived STEC isolates. No statistical difference in accessory genes was found between beef- and mutton-sourced STEC isolates (Benjamini-Hochberg corrected *p*-value >0.05). MCA of pangenomes could not separate beef- and mutton-sourced isolates (data not shown).

## 4 Discussion

In this study, we investigated the prevalence and molecular characteristics of STEC in retail raw meat in Jinan city, China. Beef is the most frequently consumed meat worldwide, consumption of undercooked beef and beef products contaminated with STEC is a main source of human STEC infection (Brashears and Chaves, 2017). An earlier review indicated that the prevalence rate of O157 and non-O157 STEC ranged from 0.1 to 54.2%, and 2.4–30.0%, respectively, in ground beef (Hussein, 2007). Previous studies in China demonstrated that the prevalence of STEC in beef ranged from 11% to 68% (Bai et al., 2015; Dong et al., 2020; Koitabashi et al., 2008). However, our study showed that 7.5% of raw beef samples were contaminated with STEC, the prevalence of O157 and non-O157 STEC in this study was 1.5% and 6.0%, respectively. The difference might be due to the limited sampling scale, as well as the sampling and isolation strategies in different studies. Mutton is less frequently reported as a source of human STEC infection compared with beef, mainly because it is usually consumed well-cooked (Mughini-Gras et al., 2018), however, it has been reported as a high carriage of STEC strains (Bai et al., 2015; Brooks et al., 2001; Kumar et al., 2014; Momtaz et al., 2013). A recent study reported that the prevalence of all STEC independent of serotype in raw ovine meat was 2.7–35.5% (McCarthy et al., 2021). Similarly, we observed that 26.6% of raw mutton samples in this study were positive for STEC. These data highlight that raw mutton/ovine meat can be important vehicle for STEC transmission. Mutton is softer and has higher free water than other meats, which may contribute to a higher capacity for proliferation and survival of microorganisms including STEC (Momtaz et al., 2013). Other factors like animal age and seasonality have been reported to affect pathogen shedding, with younger animals typically reported as having a higher prevalence of the pathogen (McCarthy et al., 2021). However, in this study these data are unavailable.

O157:H7 has been considered as the most virulent serotype associated with severe disease such as HUS (Hua et al., 2021; Ylinen et al., 2020). Two O157:H7 isolates were recovered from one mutton and one beef sample, respectively, in this study. In addition to *stx2c*, the two O157:H7 isolates carried virulence genes encoding intimin (*eae*), enterohemorrhagic *E. coli* hemolysin (*ehxA*), heat-stable enterotoxin 1 encoding gene *astA*, all of which have been reported to be associated with severe clinical outcome (Matussek et al., 2017; Schwidder et al., 2019). One mutton-sourced O26:H11 isolate in this study carried *stx1a*, *eae*, *ehxA*, and *efa1*. O26:H11 STEC strains carrying *stx1a*, *eae*, *ehxA*, and *efa1* have been reported in HUS cases (Hua et al., 2021; Mellmann et al., 2008). In addition, *ehxA* and *astA* was present in 60% and 35% of non-O157 STEC isolates, respectively. Of note, the recently-reported Stx2k subtype (Yang et al., 2020) was identified in three mutton-sourced STEC isolates. It is notable that Stx2k-STEC strains have circulated in diverse sources in China, including patients with diarrhea, and have not yet been reported in other countries. Stx2k was functional and cytotoxic to Vero cells (Yang et al., 2020). These results indicated the pathogenic potential of the meat-derived STEC isolates in this region.

Although mutton is less associated with human STEC disease compared with beef, our study showed no difference in virulence genes or accessory genes between mutton- and beef-sourced isolates. Whole genome phylogeny and MCA of pangenomes showed no separate cluster between mutton- and beef-sourced isolates either, indicating the similar genetic background of strains from different meats. It is noteworthy that the mutton-sourced O157:H7 and O26:H11 isolates in this study carried important virulence genes, and clustered with strains isolated from HUS patients, suggesting the pathogenic potential of STEC strains in this study. It has been indeed reported that mutton-sourced STECs were associated with an HUS outbreak in Norway (Schimmer et al., 2008). Given the high prevalence rate of STEC in retail raw mutton in this region, attention should be paid to food regulation and hygiene management to eliminate cross-contaminations of STEC among different foodstuffs and transmission to humans through food vehicles.

Antimicrobial resistance is a global concern for public health. Antibiotic-resistant *E. coli* strain can spread from foodstuffs to humans, we therefore examined the antimicrobial susceptibility of meat-derived STEC isolates. We found that one mutton-derived STEC isolate was resistant to three antibiotics, i.e., tetracycline, chloramphenicol, trimethoprim-sulfamethoxazole, and one mutton-derived isolate was resistant to tetracycline, the two isolates carried the corresponding resistant genes. We didn't observe antibiotic-resistant strains from beef, this may be due to the very small number of beef-sourced strains in this study, or differences in treatment of food-producing animals and age of animals at slaughter.

This study has limitations. The major flaws were the small sample size and limited sampling sites in one region. In addition, the animal age and seasonality, which are potential factors influencing STEC shedding, were unavailable in this study. Further investigation with larger samples size in different geographic locations, and epidemiological data of meats and meat-producing animals are warranted.

To conclude, this study reported high contaminations of STEC in retail raw meats, especially mutton, in Jinan city, China. Genomic characterization indicated genetic diversity of meat-derived STEC strains and their pathogenic potentials. In particular, the highly virulent serotypes O157:H7 and O26:H11 carrying the important virulence genes were identified in meat-derived STEC isolates. Additionally, the identification of mutton-derived Stx2k-STEC strains in this study suggested a wide distribution of this newly-identified Stx subtype in China, its public health risk should thus be noted. Our study highlighted the potential risk of human STEC infection through the consumption of raw meats or cross-contamination of meat-derived products. Coordinated action is therefore required to eliminate the risk of human STEC infection at different stages in food chain.

## Funding

This study was supported by the 10.13039/501100001809National Natural Science Foundation of China (82072254), and the 10.13039/501100018537National Science and Technology Major Project (2018ZX10201001-006).

## CRediT authorship contribution statement

**Bin Hu:** Conceptualization, Data curation, Methodology, Investigation, Writing – review & editing. **Xi Yang:** Methodology, Investigation, Software, Visualization, Writing – original draft, Writing – review & editing. **Qian Liu:** Methodology, Investigation, Writing – review & editing. **Yuanqing Zhang:** Methodology, Investigation, Writing – review & editing. **Deshui Jiang:** Methodology, Investigation, Writing – review & editing. **Hongbo Jiao:** Methodology, Investigation, Writing – review & editing. **Ying Yang:** Methodology, Investigation, Writing – review & editing. **Yanwen Xiong:** Investigation, Validation, Supervision, Writing – review & editing. **Xiangning Bai:** Methodology, Software, Visualization, Writing – original draft, Supervision. **Peibin Hou:** Conceptualization, Data curation, Investigation, Writing – review & editing, Supervision.

## Declaration of competing interest

The authors declare that they have no known competing financial interests or personal relationships that could have appeared to influence the work reported in this paper.

## Figures and Tables

**figs1:**
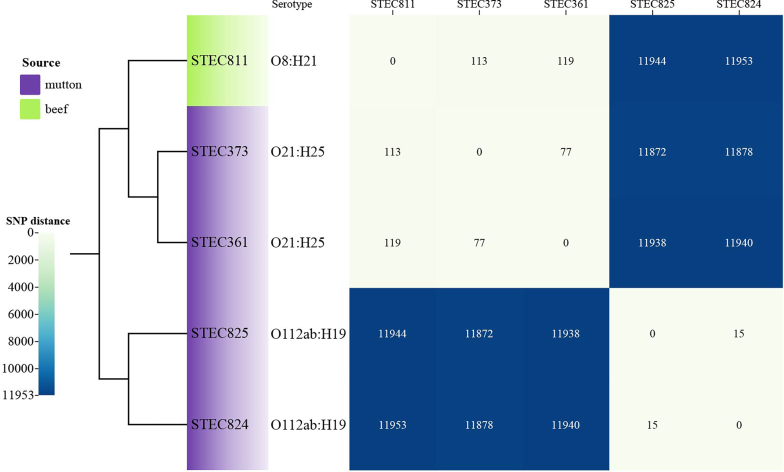
Phylogenetic tree based on single-nucleotide polymorphisms (SNPs) using the Maximum-Likelihood method. Branch length is ignored for better visualization. The source and serotype of strains are shown. The heatmap illustrates pairwise SNP distances between the STEC isolates.
